# Saline Injections for the Cure of Drunkenness

**Published:** 1852-06

**Authors:** 


From the Journal de Medecine et de Chirurgie.

*Saline Injections for the Cure of Drunkenness.*

L'ABEILLE MEDICALE has lately published a note by Dr. Lalaux, on the efficacy of injections of solution of culinary salt in rapidly dissipating the most serious symptoms of intoxication. M. Lalaux's enema consists of two good tablespoonsful of salt dissolved in four glasses of warm water. It causes a formidable shock (debacle), after which all the functions resume their play. This remedy has the advantage over ether and ammonia, of being always at hand; and, in a case of drunkenness, we had lately occasion to observe that it is more powerful than ammonia, in causing the cessation of the coma which succeeds intoxication by alcohol.

